# FGF21: An Emerging Therapeutic Target for Non-Alcoholic Steatohepatitis and Related Metabolic Diseases

**DOI:** 10.3389/fendo.2020.601290

**Published:** 2020-12-14

**Authors:** Erik J. Tillman, Tim Rolph

**Affiliations:** Akero Therapeutics, South San Francisco, CA, United States

**Keywords:** non-alcoholic steatohepatitis, nonalcoholic fatty liver disease, obesity, insulin resistance, metabolic syndrome, metabolic disease, fibroblast growth factor 21

## Abstract

The rising global prevalence of obesity, metabolic syndrome, and type 2 diabetes has driven a sharp increase in non-alcoholic fatty liver disease (NAFLD), characterized by excessive fat accumulation in the liver. Approximately one-sixth of the NAFLD population progresses to non-alcoholic steatohepatitis (NASH) with liver inflammation, hepatocyte injury and cell death, liver fibrosis and cirrhosis. NASH is one of the leading causes of liver transplant, and an increasingly common cause of hepatocellular carcinoma (HCC), underscoring the need for intervention. The complex pathophysiology of NASH, and a predicted prevalence of 3–5% of the adult population worldwide, has prompted drug development programs aimed at multiple targets across all stages of the disease. Currently, there are no approved therapeutics. Liver-related morbidity and mortality are highest in more advanced fibrotic NASH, which has led to an early focus on anti-fibrotic approaches to prevent progression to cirrhosis and HCC. Due to limited clinical efficacy, anti-fibrotic approaches have been superseded by mechanisms that target the underlying driver of NASH pathogenesis, namely steatosis, which drives hepatocyte injury and downstream inflammation and fibrosis. Among this wave of therapeutic mechanisms targeting the underlying pathogenesis of NASH, the hormone fibroblast growth factor 21 (FGF21) holds considerable promise; it decreases liver fat and hepatocyte injury while suppressing inflammation and fibrosis across multiple preclinical studies. In this review, we summarize preclinical and clinical data from studies with FGF21 and FGF21 analogs, in the context of the pathophysiology of NASH and underlying metabolic diseases.

## Introduction

A marked rise in the global prevalence of obesity and associated metabolic pathologies has been observed over the past 30 years (1). The burden of managing a chronic excess of energy over demand falls on the metabolic organs, including adipose tissue, pancreas, and liver. Ultimately the capacity of these organs to manage the surfeit of energy is exceeded, manifesting as hyperlipidemia/dyslipidemia, type 2 diabetes, and nonalcoholic fatty liver disease (NAFLD), defined as >5% liver fat content not attributable to other chronic liver conditions.

Globally, more than 1 billion people are estimated to have NAFLD, including 83 million patients in the United States, making NAFLD the most common chronic liver disease in the US (2, 3). Fifteen to twenty percent of patients with NAFLD progress to the more severe nonalcoholic steatohepatitis (NASH) (4), characterized by hepatocyte stress, injury and apoptosis, inflammation and fibrosis (5). Scarring of the liver (i.e., fibrosis) occurs in response to chronic injury and inflammation. Left unaddressed, NASH fibrosis may advance to cirrhosis, which in turn may cause end-stage liver disease or hepatocellular carcinoma (HCC) (6). The prevalence of NASH, and in particular severe F3 and F4 fibrosis, is rapidly increasing in the US and globally (7). By 2030, there are predicted to be 27 million NASH patients in the US, including 3 million with NASH cirrhosis (8). As fibrosis progresses, the risk of liver-related morbidity and mortality increases (9). NASH-related liver failure and HCC have become leading causes of liver transplantation (10, 11), particularly with the emergence of effective treatment for hepatitis C. Besides the widely recognized morbidities, pre-cirrhotic NAFLD and NASH also negatively impact health-related quality of life, further underscoring the need for effective treatments (12).

NASH is diagnosed histologically, based on the extent of steatosis, hepatocyte stress and damage (hepatocyte ballooning), immune infiltration (lobular inflammation), and degree of fibrosis (13). Within the liver, lipotoxicity-induced endoplasmic reticulum (ER) stress- and oxidative stress-induced tissue injury (14) release pro-inflammatory damage-associated molecular patterns (DAMPs) that recruit and activate Kupffer cells, liver-resident macrophages (15). Kupffer cells amplify the cycle of necroinflammation (16) while directly and indirectly activating hepatic stellate cells (HSCs) to differentiate and proliferate to a myofibroblast-like, collagen-producing phenotype that promotes fibrogenesis in NASH (17). Liver damage and ensuing inflammation may also contribute, at a whole-body level, to metabolic dysfunction in other organs and tissues (18). An ideal disease-modifying therapeutic agent needs to target the primary driver of NASH: elevated intrahepatocyte fat deposition. By removing the underlying pathogenic insult, hepatocyte oxidative and ER stress and lipotoxicity will diminish, resulting in less activation of pathways driving hepatocyte dedifferentiation and death, in turn allowing the downstream sequelae of liver inflammation and fibrosis to resolve.

While late-stage NASH greatly increases the risk of liver-related morbidity, NAFLD and NASH with less advanced fibrosis (i.e., F0–F2) are associated with greatly increased risk of cardiovascular disease and related mortality (19). Indeed, the leading cause of death among patients with NASH is cardiovascular disease, even after correcting for the contribution of other known CV risk factors and metabolic comorbidities (20–22). These highly prevalent metabolic comorbidities, including hypertriglyceridemia (83% of NASH patients), obesity (82%), dyslipidemia (72%), metabolic syndrome (71%), and type 2 diabetes (44%), underscore the critical need to holistically address the imbalanced metabolic state underlying NASH (23).

Despite the unmet medical need, there is currently no approved therapy for NASH. Lifestyle modifications, including diet and exercise, that lead to significant weight loss have demonstrated clinical benefit, but long-term compliance is poor (24, 25). The first wave of therapeutic approaches in NASH targeted downstream pathologies, specifically necroinflammation and fibrosis, either inadequately addressing or in some cases exacerbating the underlying drivers of NASH pathology: steatosis, insulin resistance, and lipotoxicity. Among numerous mid-to-late-stage failures across a variety of mechanisms of action (26–29), the only positive interim phase 3 readout in the last decade has been a trial of the bile acid analog, obeticholic acid (30), implying therapeutic target selection has been suboptimal. Moreover, none of the therapeutics in development has demonstrated both fibrosis improvement and resolution of NASH in a phase 3 clinical study, the two histological endpoints accepted by the FDA and EMA as sufficient to support accelerated or expedited regulatory approval. While the shift to address the underlying drivers of NASH holds promise, many of the mechanisms under investigation are limited to the liver as their site of action, because of safety constraints arising from systemic exposure (**Table 1**). As a consequence, these mechanisms have not improved whole-body metabolism, and in some cases have been associated with adverse effects such as increases in serum low-density lipoprotein (LDL)-cholesterol (30–32), serum triglycerides (33, 34), and body weight (35, 36).

**Table 1:** Safety and tolerability across various mechanisms of action under investigation in NASH.

Mechanism of action	Tolerability concern	Safety concern
FXR agonism	Pruritis	LDL-C increase Drug-drug interactions Hepatic decompensation
TR-β agonism	Gastrointestinal	Hypothyroidism Drug-drug interactions
PPAR agonism	Fluid retention Weight gain	Bone resorption and fractures Heart failure Cancer risk
GLP-1R agonism	Gastrointestinal	Pancreatitis Thyroid carcinoma
ACC inhibition	Gastrointestinal	Triglyceride increase Thrombocytopenia
FGF19 analog	Gastrointestinal	LDL-C increase
FGF21 analog	Gastrointestinal	Bone turnover? HPA axis activation?

FXR, Farnesoid X receptor; TR-β, Thyroid hormone receptor-β; PPAR, Peroxisome proliferator-activated receptor; GLP-1R, Glucagon-like peptide-1 receptor; ACC, Acetyl-CoA carboxylase; FGF, Fibroblast growth factor; LDL-C, Low-density lipoprotein-cholesterol; HPA, hypothalamus–pituitary–adrenal.

On the other hand, endocrine fibroblast growth factor (FGF) analogs have emerged as a promising class because of their ability to not only act directly on liver, but also to shift whole-body metabolism to a healthier state. The FGF19 subfamily of FGFs, consisting of FGF19, FGF21, and FGF23, is characterized by reduced affinity for heparan sulfate (HS) (37). In the absence of HS binding, these FGFs may diffuse from their site of secretion, enabling endocrine actions *via* the systemic circulation, as well as canonical FGF paracrine signaling on their cell of origin or nearby cells (38). Whereas HS in extracellular matrix serves as a coreceptor for autocrine and paracrine FGFs, the endocrine FGFs rely on high-affinity interactions with either α-Klotho (FGF23) or β-Klotho (FGF19 and FGF21) for recruitment and localization to the cell surface, where they engage canonical FGF receptors (FGFRs) (39–42). Dysregulation of endocrine FGF signaling, particularly FGF19 and FGF21, has been implicated in metabolic disease, suggesting their potential as therapeutic mechanisms (43–46). While gut-secreted FGF19 and liver-secreted FGF21 (47) share some overlapping physiological roles, including regulation of glucose and lipid metabolism (48), significant differences exists between them, primarily in the ability of FGF19 to stimulate hepatocyte proliferation and to suppress bile acid synthesis (49). These divergent actions result from different agonist profiles across the FGFRs: whereas the FGF19/β-Klotho receptor complex is able to bind and activate FGFR1c, FGFR2c, FGFR3c, and FGFR4, the FGF21/β-Klotho complex signals only through FGFR1c, FGFR2c, and FGFR3c (41).

This review examines the unique physiology of FGF21 signaling at the cellular, tissue, and whole-body level, presenting preclinical and clinical evidence that FGF21-based therapeutics exert a broad set of metabolic actions and also suppress inflammation and fibrosis. In particular, the discussion focuses on the promise of FGF21 analogs as foundational treatments for NASH.

## Metabolic Dysregulation Underlying Early Steatohepatitis Pathology

**Figure 1:**
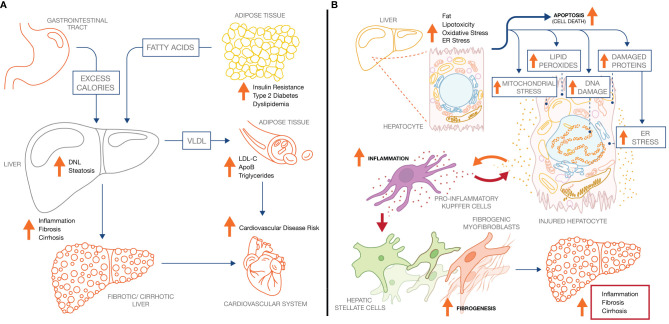
NASH pathology and pathophysiology. **(A)** Increased adipose tissue lipolysis-derived fatty acid flux and increased hepatic DNL drive hepatic steatosis in NASH, which increases both liver-related mortality and cardiovascular mortality. **(B)** Increased intrahepatic fat drives pathophysiological processes across the hepatocyte, inflammatory Kupffer cell, and collagen-producing hepatic stellate cell. Ultimately, continuous exposure to the underlying stressors (metabolic dysregulation, lipotoxicity) sustain the inflammatory and fibrotic phenotype leading to progressive fibrosis and cirrhosis.

Insulin resistance, obesity, and dyslipidemia are the clinical manifestations of a chronic excess of energy. To reduce energy uptake, peripheral organs including adipose tissue and skeletal muscle respond by reducing sensitivity to insulin. As a result, dietary energy is diverted to the liver, leading to excessive accumulation of lipid droplets (LD) in hepatocytes (hepatic fat fraction of up to 20–30%, vs <5% in healthy liver) (5), and hyperlipidemia (50). In NASH patients, the sources of hepatic fatty acid flux are presented in **Figure 1A**: 45–55% is accounted for by adipose tissue lipolysis; 25–35% by *de novo* lipogenesis (DNL) in the liver; and 10–20% by dietary fat (51, 52). Impaired peripheral insulin sensitivity (52) contributes substantially to the increased flux of fatty acid in liver. Post prandial suppression of adipose tissue lipolysis by insulin is impaired (48), while uptake by adipose tissue of dietary fat as chylomicrons is also attenuated. Insulin-dependent glucose uptake by adipose tissue and skeletal muscle is likewise reduced, redirecting glucose to the liver where it fuels DNL by activating the carbohydrate response element-binding protein (ChREBP), a master transcriptional regulator of lipogenic pathway proteins (53). ChREBP is also induced in liver by dietary fructose, a major source of calories in “Western” obesogenic diets (54, 55). Increased esterification of fatty acid by liver is associated with higher secretion of triglyceride into the circulation as VLDL. Accumulation of fat in the circulation manifests as elevated serum triglyceride and LDL-cholesterol, in combination with low levels of high-density lipoprotein (HDL), characteristic of the dyslipidemia prevalent in NASH patients (56).

Chronic accumulation of triglyceride in liver elevates the basal rate of fatty acid β-oxidation in hepatocytes. Conditions in which energy supply from fat oxidation exceeds energy demand lead to formation of reactive oxygen species, in turn causing oxidative stress (57, 58). This stress, which damages hepatocytes, also induces transcription and secretion of FGF21, which appears able to act directly on hepatocytes as a paracrine or autocrine hormone (59, 60).

Preclinical characterization of FGF21 in rodents and primates has established its role in restoring metabolic homeostasis in obese or metabolically challenged animals, reducing body weight, liver and circulating triglycerides, fasting plasma insulin and glucose, and increasing energy expenditure (43, 61). Consistent with a protective role, endogenous FGF21 serum concentration may be elevated by up to 10–20-fold in patients with NAFLD (62, 63), NASH (64), obesity (64–66), type 2 diabetes (67), chronic kidney disease (68), diabetic nephropathy (69), atherosclerosis (70, 71), or coronary heart disease (72). However, this elevation of FGF21 observed in chronic, pathological metabolic states in humans does not appear sufficient to ameliorate disease.

While decreased FGF21 receptor expression has been proposed to underlie this “FGF21-resistant state,” (73), the dramatic upregulation of fibroblast activation protein (FAP), an endopeptidase that cleaves and inactivates endogenous FGF21 (74), has been reported in liver and serum of patients with metabolic liver disease (75–77). Together, these observations have guided the design and development of FGF21 analogs that may restore metabolic homeostasis. The physiology and dysregulation of FGF21 signaling in NASH is discussed in detail below, particularly in the context of FGF21 resolving discrete aspects of NASH pathology.

## Fibroblast Growth Factor 21 Physiology

FGF21 relies on its obligate co-receptor, β-Klotho, for recruitment to the extracellular surface of plasma membrane (78). The β-Klotho/FGF21 receptor complex interacts specifically with the cognate receptors: FGFR1c, FGFR2c, or FGFR3c (40, 41), enabling downstream FGFR signaling *via* pathways including the mitogen-activated protein kinase (MAPK) and AKT signaling networks (79).

### Endocrine Role of Fibroblast Growth Factor 21 Physiology

**Figure 2:**
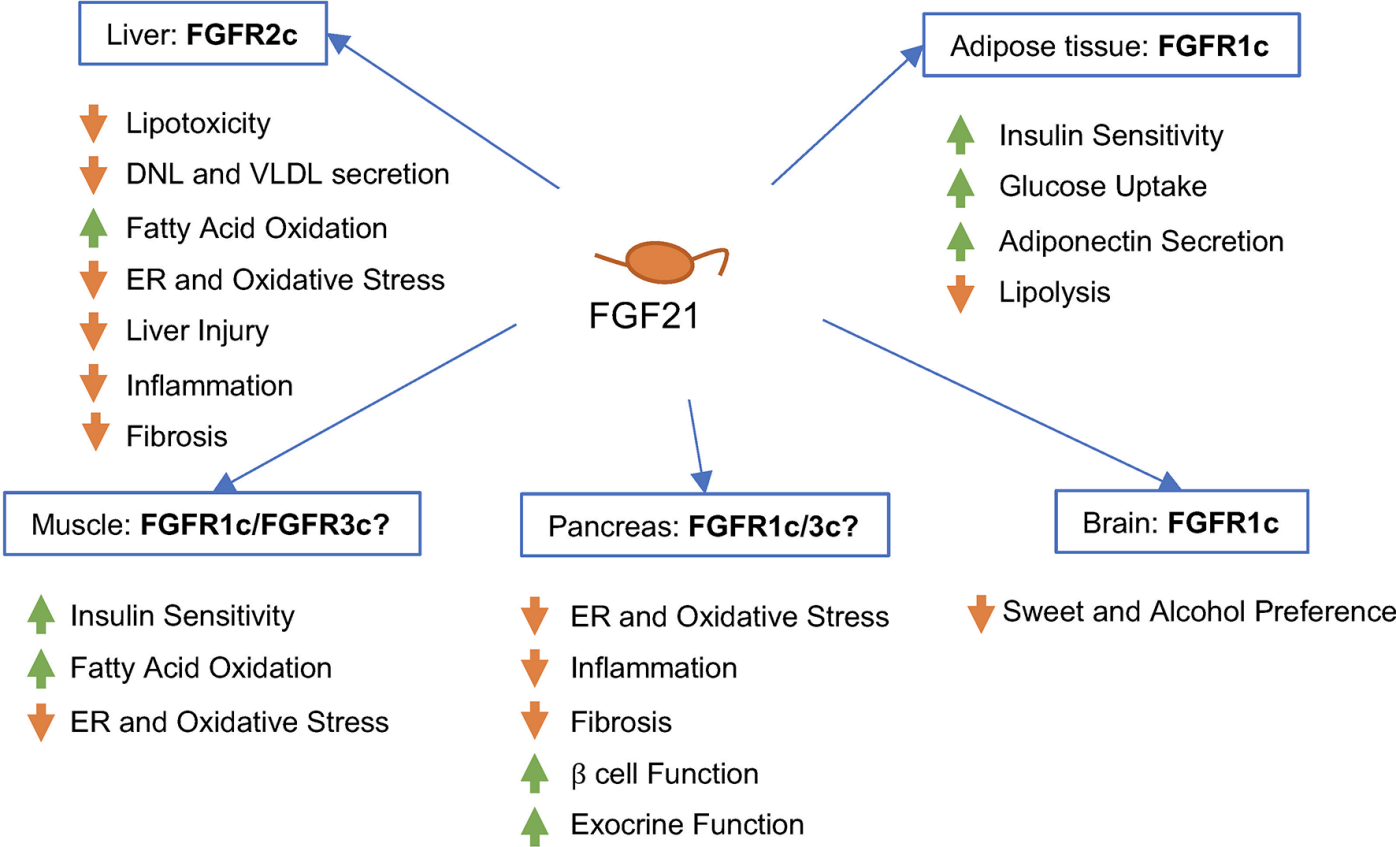
Predicted effects of FGF21 signaling in different human tissues and cell types *in vivo*. FGF21 binds -Klotho on the cell surface, enabling interaction of the ligand/receptor complex with the FGFR isoform present on that cell type. FGF21 signaling appears to be primarily mediated by FGFR1c in adipose tissue, FGFR2c in liver, and FGFR1c or FGFR3c in pancreas. The contribution of FGFR1c signaling in the brain to the metabolic effects of FGF21 in humans remains to be elucidated.

Because FGF21 enters the systemic circulation, it is able to integrate metabolism across liver, adipose tissue, skeletal muscle, pancreas, and other metabolic organs (**Figure 2**), by controlling expression of transcriptional programs that shape cellular phenotype and tissue metabolic function, ultimately exerting anti-obesity, anti-diabetic, and anti-hyperlipidemic effects in rodents and primates (43, 44).

Expression of FGF21 is regulated by metabolic cues. Changes in organismal nutritional state, such as those induced by fasting or starvation (80), high-carbohydrate diets (81, 82) or low-protein diets (83–86) activate FGF21 expression. These diverse states activate transcription from the *FGF21* locus *via* well-characterized nutritional and metabolite-responsive transcription factors, including fatty acid-responsive nuclear hormone receptors peroxisome proliferator-actived receptor- (PPAR)α and PPARγ (87–92), and the glucose and lipid homeostasis-controlling transcription factor CREBH (93, 94).

FGF21 appears to be mainly synthesized in the liver (95), with dynamic contributions from the pancreas, adipose tissue, and skeletal muscle (96). The target tissues of FGF21’s endocrine actions are determined by overlapping tissue expression patterns for β-Klotho and cognate FGFRs.

In mice, β-Klotho is primarily expressed across metabolic tissues including liver, pancreas, and adipose tissue (96, 97). FGFR1c is much more highly expressed than other FGFR isoforms in adipose tissue and pancreas (41, 73, 96, 98, 99). FGFR2c is more highly expressed than FGFR1c or FGFR3c in liver (96, 100). *FGFR2c* and *KLB* (the gene encoding β-Klotho) are induced upon exposure to FGF21 or an FGF21 analog, consistent with enhanced sensitivity to FGF21’s metabolic effects (101).

In humans, FGFR1c is also the predominantly expressed receptor in adipose tissue, with mRNA levels up to two orders of magnitude higher than for *FGFR2c* or *FGFR3c* (99, 102). *FGF21, FGFR1c*, *FGFR2c*, and *FGFR3c* are also expressed in skeletal muscle (103, 104). *FGFR2c* and, to a lesser extent, *FGFR3c* are expressed basally in liver (102), and *FGFR2c* is more highly expressed in NAFLD relative to healthy liver (99). Obesity increases expression of *FGF21*, *KLB*, *FGFR1c*, and *FGFR3c* in the liver, although FGFR2c remains the most highly expressed of FGF21’s receptors in the liver. In contrast, obesity reduces the expression of *KLB* in subcutaneous and visceral adipose tissue (102).

As an endocrine factor, FGF21 has been shown in preclinical models to exert effects in diverse tissues beyond liver, including adipose tissue, pancreas, skeletal muscle, and the central nervous system. These diverse extrahepatic actions provide broad metabolic benefits and help to resolve hepatic steatosis in patients with NASH. In particular, agonism of FGFR1c appears essential to mediating FGF21’s suppression of fatty acid flux from adipose tissue to liver. On the other hand, direct modulation of metabolism and mediation of FGF21’s cytoprotective effects in liver, described below, likely requires agonism of FGFR2c and possibly FGFR3c. Consequently, analogs of FGF21 designed for treating NASH should posess balanced agonism across FGFR1c, FGFR2c, and FGFR3c.

### Integration of Endocrine Actions With Other Hormones

The interaction of FGF21 with other hormones that regulate metabolism is complex. On one hand, FGF21 appears to increase peripheral anabolic signaling, for example through muscle and adipose tissue insulin sensitization, under conditions of adequate or excessive energy intake, as described in detail below. On the other hand, FGF21 appears to augment catabolic hormone signalling under conditions of inadequate energy intake, for example by increasing adrenal gland sensitivity to adrenocorticotropic hormone by upregulating levels of steroidogenic proteins (105). FGF21 also appears to mediate some of the effects of hepatic glucagon and thyroid hormone signaling, since both activation of glucagon receptor (GCGR) (106) and thyroid hormone receptor-β (TR-β) (107) induce secretion of FGF21 by hepatocytes. The cooperative interaction between FGF21, glucagon and thyroid hormone would be expected to increase glucose output and oxidation of fat by liver (108, 109). In the setting of NASH, increasing liver fat oxidation should help reduce steatosis, while exporting energy from the liver as glucose aids in unburdening the liver of excess energy. Simultaneously, the released glucose is taken up more effectively by peripheral organs due to FGF21-enhanced insulin sensitivity, described in detail below.

### Autocrine/Paracrine Role of Fibroblast Growth Factor 21 Physiology

Expression and secretion of FGF21 is activated in many cell types experiencing ER stress and oxidative stress. FGF21 induces a number of pathways that serve to protect cells against these stressors, while simultaneously inhibiting pro-cell death pathways. Mitochondrial dysfunction leading to oxidative stress also induces FGF21 expression in preclinical disease models (110), while levels of FGF21 are elevated in the circulation and skeletal muscle of patients with myopathies caused by mutations in components of the mitochondrial oxidative phosphorylation machinery (111).

Enhanced secretory load, imbalances in redox homeostasis, or perturbations in protein homeostasis triggered by lysosomal or autophagosomal dysfunction may all cause ER stress and trigger the unfolded protein response (UPR). Across multiple tissues, FGF21 appears to counteract ER stress (101, 112, 113).

## Potential of Fibroblast Growth Factor 21 Physiology as A Therapy For Non-Alcoholic Steatohepatitis

### Steatosis and Lipotoxicity in Non-Alcoholic Steatohepatitis

Steatosis in hepatocytes is characterized microscopically by accumulation of triglyceride in numerous cytosolic LDs. Recent studies of human genetic associations with NAFLD and NASH have confirmed the central role of aberrant LD biology in disease etiology. Three risk alleles uncovered in genome-wide association studies on NASH patients include polymorphisms in the LD-associated genes *HSD17B13* (114), *PNPLA3* (115), and *TM6SF2* (116). These risk alleles correlate with increased rates of liver injury, fibrosis, HCC, and liver-related mortality. How these genotypes influence LD homeostasis is the subject of considerable ongoing research. The pathogenic variant of *TM6SF2* increases the risk and severity of NASH but reduces cardiovascular disease, seemingly by reducing triglyceride secretion from the liver as VLDL (117). While accumulation of esterified triglyceride in hepatocyte LDs are not directly lipotoxic, their constant turnover releases lipotoxic species including fatty acids, diacylglycerol, free cholesterol and ceramide (118–120). Saturated fatty acids liberated from LDs, or taken up from circulation into the liver, may directly promote tissue injury, leading to additional macrophage recruitment and activation (121). Unesterified fatty acids also destabilize lysosomes leading to activation of NF-κB-dependent tumor necrosis factor α (TNF-α) expression which potentiates lipotoxicity-driven inflammation (122). Palmitate, a major saturated fatty acid, has also been reported to contribute directly and indirectly to proteotoxic stress and ER stress (123). Unsaturated fatty acids under conditions of oxidative stress form highly reactive epoxides resulting in formation of protein adducts (124), which also increase ER stress (125).

#### Fibroblast Growth Factor 21 Reduces Hepatic Steatosis and Lipotoxicity

Administration of FGF21, FGF21 analogs or adenoviral delivery of FGF21 reduces hepatic steatosis in diverse rodent models of NAFLD and NASH (101, 126–129). The reduction in liver fat results from pleiotropic actions of FGF21, particularly suppressing caloric burden in the liver, reducing *de novo* lipogenesis, and increasing fat oxidation in the liver. In diet-induced obese (101, 129) or non-obese (130) mice, FGF21 or an FGF21 analog reduces expression of lipogenic genes, including *SCD1*, *FASN*, and/or *SREBF1*, a master regulator of the lipogenic transcriptional network that is overexpressed on a high-fat diet. The reduction in lipogenic gene expression appears attributable to both weight loss and mechanisms independent of weight loss, since a similar magnitude of weight loss induced by a low-calorie diet elicited a smaller reduction in lipogenic gene expression than FGF21 (101). Conversely, an FGF21 analog increases expression of genes involved in mitochondrial β-oxidation of fatty acids (131). The reduction in hepatic steatosis by FGF21 is independent of AMPK-dependent inhibition of acetyl CoA carboxylase (ACC) (132).

### Oxidative and Endoplasmic Reticulum Stress in Non-Alcoholic Steatohepatitis

Another of the genetic variants associated strongly with NASH, the I148M variant of patatin-like phospholipase domain-containing protein 3 (PNPLA3) appears to disrupt release of mono-unsaturated fatty acids (MUFAs), which activate peroxisome proliferator-activated receptor gamma coactivator 1-α (PGC1α) and nuclear factor erythroid 2-related factor 2 (NRF2). These transcription factors mediate increases in fatty acid oxidation capacity and pathways protective against oxidative stress (86–88). NADPH oxidase 4 (NOX4), a ROS-producing enzyme, is highly expressed in the livers of NASH patients and contributes to pathological inflammation and fibrosis in a mouse model of diet-induced NASH (94). Chronic oxidative stress may exacerbate LD accumulation as a means to regenerate NAD from NADH under conditions of excess energy supply (**Figure 1B**), as observed in cultured mouse hepatocytes in which expression of fatty acid synthase (*FASN*) and sterol response element-binding protein 1c (*SREBF1*) are induced (133). Moreover *in vivo* knockout of genes encoding antioxidant enzymes SOD1 or GPX1 induces hepatic expression of SREBP1c and SREBP2, and increases liver triglyceride content by over 50% (134).

An increase in oxidative species and altered redox leads to an accumulation of misfolded or damaged proteins and in turn ER stress (135). In healthy hepatocytes, autophagy degrades damaged proteins, but this pathway appears disrupted in liver of patients with NAFLD (98). Stabilization and activation of mammalian target of rapamycin complex 1 (mTORC1) by acetyl CoA, the product of fatty acid oxidation, may contribute to disruption of autophagy (136).

The UPR has three canonical branches (PERK/ATF4, IRE1a/XBP1, and ATF6), which play a central role in mitigating cellular stress by enhancing protein folding capacity and secretion, activating the oxidative stress response, and inducing autophagy, thereby restoring protein homeostasis (137). In preclinical studies, UPR dysregulation contributes to oxidative damage and hepatocyte death in a mouse model of fructose-induced NAFLD (138). UPR function also appears compromised in maintaining redox balance within hepatocytes in NAFLD and NASH patients (139). Two branches of the UPR appear, at least in part, to suppress steatosis in animal models (140). Loss of either the ATF6 or the IRE1α pathways sensitizes animals to ER stress-induced liver steatosis, metabolic dysregulation and mortality (140). Ablation of either of these branches increases hepatic lipogenic gene expression and steatosis while leading to unresolved ER stress, characterized by sustained UPR activation and, in particular, elevated ATF4/CHOP signaling. One potential mechanism underlying this steatosis may be upregulation of hepatic VLDL receptor expression by PERK/eIF2α signaling, thereby enhancing hepatocyte triglyceride uptake (141). In addition to contributing to the steatotic drive associated with sustained UPR signaling (140), CHOP mediates the pro-apoptotic effects of the UPR in the post-adaptive phase (137).

Unresolved lipotoxicity, oxidative stress, and ER stress ultimately trigger apoptosis of hepatocytes (**Figure 1B**). Apoptotic cell death is observed both in liver and in peripheral tissues in NAFLD patients (142, 143). CHOP drives hepatocyte apoptosis in part *via* transcriptional regulation of autophagy (144). Toxic lipids may themselves stimulate apoptosis *via* IRE1α-dependent activation of the pro-apoptotic c-Jun N-terminal kinase, JNK1, a MAPK (145). Intracellular free cholesterol levels, which trigger apoptosis of hepatocytes in a JNK1-dependent manner, are elevated in NAFLD patients, correlating with degree of histological disease activity (146). Pro-apoptotic signaling of JNK1 is mediated by the upstream MAP3K apoptosis signal-regulating kinase (ASK1) cascade (147). Apoptotic and lipo-apoptotic cell death is dependent on mitochondrial disruption by pro-apoptotic mediators (e.g., BAX, BAD, etc) that in turn release molecules that activate caspase-mediated degradation and cell death. Upon cell death, hepatocytes release DAMPs, the degradation products of endogenous subcellular structures and proteins that recruit and activate Kupffer cells, thereby initiating and progressing inflammatory liver disease (148).

#### Fibroblast Growth Factor 21 Reduces Oxidative Stress and Endoplasmic Reticulum Stress

Expression and secretion of FGF21 is induced by oxidative stress. For example, blocking hepatic oxidation of long-chain fatty acids induces oxidative stress and expression of FGF21 (149). Furthermore, acute oxidative stress induced by acetaminophen markedly upregulates expression of FGF21, β-Klotho and antioxidant enzymes in hepatocytes (150). Acting as an autocrine agent, FGF21 activates pathways that protect against oxidative stress. Consistent with potent antioxidant effects in liver, FGF21 protects mouse liver from acetaminophen-induced oxidative damage (150). In wild-type mice, FGF21 treatment increases hepatic transcription of the canonical oxidative stress response and antioxidant genes *Sod2* (superoxide dismutase 2), *Cat* (catalase), *Gpx1* (glutathione peroxidase 1), *Sirt1* (sirtuin), and *Foxo3* (forkhead box transcription factor 3) (151). In liver of obese diabetic mice, FGF21 reduces oxidative damage and lipid peroxidation (152).

FGF21 is also induced by the UPR. In diet-induced obese mice and in NAFLD patients, activation of all three branches of the UPR is observed, with increased nuclear ATF4, ATF6, and XBP1 protein levels along with increased whole-lysate phosphorylated eIF2α, phosphorylated IRE1α, and FGF21 (153). In mice, this induction of FGF21 expression requires IRE1-XBP1 signaling. Acting as an autocrine or paracrine hormone, FGF21 appears both to induce pathways that restore protein homeostasis, for example by enhancing TFEB-dependent lysosomal biogenesis to enable turnover of damaged proteins (154, 155); and also to act as a negative feedback loop to suppress chronic activation of the ATF4/CHOP-mediated, pro-steatotic and pro-apoptotic pathway. The eIF2α/PERK/ATF4 branch of the UPR also activates FGF21 under different conditions of ER stress, including change in ER redox state upon dithiothreitol treatment, or perturbation of ER calcium homeostasis with thapsigargin (156). Because lipid accumulation, lipotoxicity, oxidative stress, and ER stress all appear to be fundamental drivers of NASH pathogenesis, FGF21-based therapies may be broadly useful in a potentially heterogeneous NASH patient population.

#### Fibroblast Growth Factor 21 Reprograms Hepatocyte Gene Expression

FGF21 signaling in hepatocytes may promote epigenetic reprogramming to a healthier metabolic phenotype. In mice, fasting-induced FGF21 signaling leads to PKA-dependent recruitment of a histone demethylase, JMJD3, to the promoters of autophagy genes (157). JMJD3 demethylation of these promoters de-represses target autophagy genes, which in turn restores functional autophagy and lipid catabolism in obese animals. In fibrotic and cirrhotic liver tissue from patients with NASH (158, 159), reduced expression of HNF4α, a master regulator of mature hepatocyte cell fate, has been observed. In high-fat diet-induced obese mice, FGF21 treatment increases hepatocyte HNF4α by a weight loss-independent mechanism (101). Moreover, transgenic overexpression of FGF21 rescues rats from liver failure by restoring a mature hepatocyte transcriptional profile to dedifferentiated hepatocytes, in turn improving their function (160).

### Inflammation and Inflammatory Signaling in Non-Alcoholic Steatohepatitis

Liver resident macrophages, known as Kupffer cells, are activated by DAMPs and pro-inflammatory cytokines released by stressed and apoptotic hepatocytes (15). DAMPs bind and activate pattern recognition receptors (PRRs) on Kupffer cells, including Toll-like receptor 4 (TLR4), which is upregulated in NASH livers, leading to nuclear translocation of a master transcriptional regulator of inflammation, nuclear factor kappa-light-chain-enhancer of activated B cells (NF-κB) (161). Mitochondrial DNA released from damaged or dying hepatocytes also activates Kupffer cells *via* TLR9 receptors (117). Transcription of NF-κB target genes activates Kupffer cells, leading to secretion of pro-inflammatory cytokines and chemokines including TNFα (162), IL-1β (163), IL-6 (164), monocyte chemoattractant protein-1 (MCP-1)/CCL2 (165), and C-X-C motif chemokine ligand-10 CXCL10 (166). These signals serve to recruit monocyte-derived macrophages, neutrophils, and T-helper cells (Th1 and Th17) to the inflammatory niche (167, 168). The resulting, locally elevated concentration of pro-inflammatory molecules directly causes hepatocyte injury and apoptosis, for example *via* TNFα-mediated death receptor activation (169), initiating and perpetuating a “vicious cycle” of necroinflammation leading to loss of hepatocytes and of the liver’s functional capacity (170).

In addition to release of PRR ligands, hepatocyte stress and death are associated with release of pro-inflammatory cytokines, including inflammasome-mediated interleukin-1β (IL-1β), which further contributes to NASH-associated hepatitis (171). Components of the NOD-, LRR- and pyrin domain-containing protein 3 (NLRP3) inflammasome are more highly expressed in liver samples from NASH patients than those without NASH (172). The NLRP3 inflammasome cleaves and activates caspase 1, which not only activates IL-1β but also IL-18 (171). In mice, inactivation of NLRP3 or inflammasome signaling suppresses inflammation and fibrosis in models of NASH (173, 174) and alcoholic steatohepatitis (175) by reducing recruitment and activation of Kupffer cells (174). NLRP3 activation in hepatocytes can also lead to caspase 1-mediated pyroptosis, or inflammasome-mediated lytic cell death, amplifying the release of DAMPs beyond just apoptotis-associated DAMP release (176).

#### Fibroblast Growth Factor-21 Exerts Direct Anti-Inflammatory Effects

FGF21 suppresses inflammation in NASH models, as well as in models of other inflammatory diseases. Consistent with a direct anti-inflammatory effect in multiple cell types, FGF21 inhibits macrophage expression of the pro-inflammatory cytokines TNFα, IL-6, IL-1β, and IFN-γ by inducing NRF2 nuclear translocation, thereby blocking NF-κB activation (177). FGF21 also reduces fatty acid-induced hepatocyte expression of TNFα and the NF-κB subunit p65, and LPS-induced hepatocyte expression of IL-6 *in vitro* (178). The broad anti-inflammatory effects of FGF21 have also been demonstrated in cell-based models of inflammatory cytokine secretion by pulmonary endothelial cells (179) and human lung cells (180).

These same inflammatory pathways are suppressed *in vivo* upon FGF21 administration in animal models of NASH and other metabolic diseases. In obese diabetic mice with elevated inflammatory signaling in liver, administration of an FGF21 analog reduces hepatic activation of NF-κB and JNK1/2, inhibiting TNFα expression and consequent macrophage recruitment (152). Conversely, loss of FGF21 increases both liver and plasma cytokine levels in mice, including TNFα, IL-6, and MCP-1, upon chronic alcohol exposure (181). Loss of FGF21 also potentiates fructose-induced hepatic expression of the inflammatory genes MCP-1/CCL2, MIP1α/CCL3, and CD68, suggesting that FGF21 maintains liver health during chronic fructose exposure (182). In liver tissue of high-fat diet-induced obese mice, FGF21 treatment augments expression of the immunosuppressive gene *IKBKE* and reduces expression of the pro-inflammatory gene *IL-18*, independent of FGF21-induced weight loss (101). Additionally, an FGF21 analog reduces expression of pro-inflammatory and increases expression of anti-inflammatory genes in non-human primate adipose tissue (183). Finally, FGF21 reduces expression and activation of the NLRP3 inflammasome in vascular endothelial cells in diabetic mice (184), which both protects the cells themselves and reduces further inflammatory damage by suppressing pyroptosis and immune cell recruitment (185).

Consistent with its suppression of pro-inflammatory gene expression, FGF21 inhibits immune cell recruitment and activation *in vivo*. An FGF21 analog reduces neutrophil and macrophage infiltration in the liver of obese non-human primates with NAFLD (186). Adenovirus-mediated long-term overexpression of FGF21 in high-fat diet-induced obese mice suppresses hepatic and adipose inflammatory cell infiltration and activation (187). FGF21 also indirectly suppresses recruitment of CD4+ Th17 cells and secretion of IL-17 in a choline-deficient, high-fat diet model of NASH, by enhancing adiponectin release from adipose tissue (128). Consistent with suppression of the Th17/IL-17 axis in NASH models, FGF21 reduces the expression of IL-17 and the expansion of Th17 cells in a mouse model of rheumatoid arthritis (188). FGF21 significantly suppresses immune infiltration and inflammatory gene expression in cerulein-induced pancreatitis in mice (189), protects against high-fat diet-induced pancreatic lymphocytic inflammation and islet dysfunction (190), and reduces macrophage infiltration in murine pancreatic ductal adenocarcinoma (191).

In summary, FGF21’s anti-inflammatory actions likely arise from both direct suppression of proinflammatory signalling, by innate and possibly adaptive immune cell types as well as attenuation of immune-effector cell infiltration into the liver, and its indirect inhibition of pro-inflammatory signals released by injured hepatocytes. Because inflammation is a core component and driver of NASH pathology, the anti-inflammatory actions of FGF21-based therapies are likely to benefit the general NASH patient population.

### Fibrosis in Non-Alcoholic Steatohepatitis

In addition to exacerbating hepatocyte injury and apoptosis, activation of Kupffer cells and expansion of the inflammatory niche stimulates differentiation of liver-resident HSCs into pro-fibrogenic myofibroblasts (192) (**Figure 1B**). In particular, hepatocyte- or Kupffer cell-derived TGF-β, platelet-derived growth factor (PDGF) and TNFα stimulate and sustain proliferation and differentiation of HSCs. HSC activation initially promotes wound healing and liver regeneration (193), including elevated production of type I and type III collagen, as well as proteases and cytokines that drive remodeling of extracellular matrix (ECM). However, chronic inflammation sustains this fibrogenic signaling in the HSC niche, leading to progressive liver fibrosis and ultimately cirrhosis (193–196). HSCs are also activated by increased attachment to the ECM, mediated by integrin/focal adhesion kinase signaling, suggesting a self-sustaining program of collagen deposition and HSC activation (197). By secreting inflammatory cytokines and cell adhesion molecules (198), activated HSCs in turn further amplify and perpetuate the inflammatory cycle by promoting macrophage activation and infiltration.

Inflammation is not the only pathogenic mechanism underlying fibrosis in livers of NASH patients, as dysregulation of a number of developmental and metabolic pathways also contributes to fibrosis (199). Notch ligands NOTCH1 and NOTCH2 and their corresponding receptors are upregulated in liver of patients with type 2 diabetes or NASH (200) compared to those without (201). Hepatocytes in particular demonstrate marked upregulation of the canonical Notch target gene, *HES1*, compared to nonparenchymal cells, correlating with disease severity (132). In a mouse model of diet-induced NASH, Notch signaling in hepatocytes induces osteopontin secretion, paracrine HSC activation and hepatic fibrosis, which are abrogated by Notch inhibition (132). Like Notch signaling components, levels of osteopontin protein and gene expression are positively correlated with NAFLD disease severity and extent of fibrosis (202).

Hepatocyte triglyceride accumulation and cholesterol dysregulation also directly drive HSC activation *via* induction of the Hippo pathway (146, 199). Marked upregulation of the Hippo pathway transcriptional activator, TAZ, is evident in liver samples from NASH patients, but not from patients with simple steatosis (149). Elevated levels of hepatocyte free cholesterol in NASH (203) stabilize TAZ, which is constitutively degraded (204). Stabilized TAZ directly activates transcription of the Hedgehog pathway ligand, Indian Hedgehog (IHH), which is secreted from hepatocytes and activates HSCs, thereby promoting collagen secretion and fibrogenesis (149). In mice, overexpression of hepatocyte TAZ is sufficient to drive NASH progression, whereas TAZ silencing attenuates inflammation and fibrosis (149). Hepatocyte Hippo pathway activity also upregulates expression of the pro-inflammatory and pro-fibrotic gene, *Cyr61/CCN1*, which is highly expressed in livers of NASH patients (205).

Stressed and injured NASH hepatocytes express and secrete a second Hedgehog ligand, Sonic Hedgehog (SHH), which is positively correlated with hepatocyte ballooning, steatosis, pericellular fibrosis, and fibrosis stage in liver samples from NASH patients (206, 207). Hepatocytes that highly express SHH appear colocalized within fibrotic areas in livers of NASH patients (208), and are proximal to areas of hepatic progenitor cells (209), consistent with the observation that SHH signaling promotes and maintains a de-differentiated liver cell state (210). SHH-positive livers from NASH patients express higher levels of fibrogenic and immunomodulatory genes compared to SHH-negative NASH liver samples, whose transcriptomes resemble steatotic, non-NASH livers (209). Decreases in SHH expression are significantly associated with improvement in histological features of NASH and reduced prevalence of hepatic progenitor cells in patients treated with vitamin E in the PIVENS trial (211). These data are consistent with a clear pro-fibrotic paracrine signal originating from the injured, ballooned hepatocyte.

In summary, the reprogramming of hepatocytes associated with a profibrotic milieu results in loss of mature parenchymal cells, characterized by loss of the master hepatocyte transcription factor HNF4α (158, 212) and upregulation of the developmental Notch, Hippo (213), and Hedgehog (214) signaling pathways. In addition to accelerating the loss of metabolically competent hepatocytes and liver function, advanced fibrosis and cirrhosis restrict hepatic blood flow, increasing the risk of acute liver failure, HCC, and liver-related mortality (215). The extensive cross-talk between liver cell types during NASH pathogenesis and progression underscores the importance of targeting multiple aspects of liver physiology and pathophysiology in treating this complex disease.

#### Fibroblast Growth Factor 21 Exerts Direct Anti-Fibrotic Effects

FGF21 and its analogs have demonstrated consistent anti-fibrotic effects in cell culture systems and animal models of NASH, liver injury, or metabolic disease. An FGF21 analog reduces expression of fibrogenic type I collagen and α-SMA in the LX-2 human HSC line induced by either succinate, palmitate, or culture medium devoid of methionine and choline (216). Consistent with these gene expression changes, FGF21 significantly reduces HSC activation and proliferation *in vitro* (216). FGF21 also inhibits ethanol- or PDGF-induced expression of type I collagen and α-SMA in the T6 rat HSC line (217).

Examining hepatic gene expression and histology *in vivo* corroborates FGF21’s direct inhibition of fibrosis arising from liver injury induced by diverse insults. For example, in obese diabetic mice fed a methionine and choline-deficient diet (MCD), an FGF21 analog significantly reduces fibrogenic expression and inhibits hepatic fibrosis (152). In the same model, FGF21 also reduces the cholesterol level in the liver, thereby suppressing activation of the Hippo pathway, which in turn attenuates Indian Hedgehog (IHH)-dependent activation of HSCs (204). In contrast, loss of FGF21 potentiates MCD-induced liver injury and fibrosis, which is prevented by administering exogenous FGF21 (218). Similarly, in an alcohol-induced model of hepatic fibrosis, loss of FGF21 exacerbates liver injury and fibrosis (219). FGF21 also inhibits fibrogenesis in two mouse models of chemically-induced liver injury, dimethylnitrosamine- (217) or thioacetamide-induced fibrogenesis (220), by blocking TGF-β expression and signaling, and NF-κB activation.

Additional evidence for FGF21’s direct anti-fibrotic action is provided by multiple models of tissue injury. FGF21 reduces cerulein-induced exocrine pancreatitis in mice by activating FGFR signaling in pancreatic acini and suppressing fibrogenic gene expression (221). Administration or overexpression of FGF21 reduces murine renal fibrosis in a diabetic model (222) and a ureteral obstruction model (223). Likewise, FGF21 administration inhibits formation of bleomycin-induced pulmonary fibrosis *in vivo* by suppressing oxidative stress *via* NRF2 activation, and inhibiting TGF-β-induced activation of pulmonary myofibroblasts (224).

FGF21’s breadth of antifibrotic effects are likely mediated both by direct suppression of HSC activation, and by reductions in expression and secretion of pro-fibrotic factors derived from Kupffer cells and injured hepatocytes. With these diverse anti-fibrotic effects, FGF21-based therapies may deliver clinically meaningful histologic improvements independent of the extent of fibrosis at baseline.

## Fibroblast Growth Factor 21 Improves Whole-Body Metabolic Health

In contrast to many candidates being clinically tested as potential NASH therapeutics, FGF21 not only exerts direct effects on the liver to improve its metabolic state, but also acts on whole body metabolism to lighten the metabolic load on the liver.

### Fibroblast Growth Factor 21 Improves Peripheral Insulin Sensitivity and Promotes Uptake of Energy by Adipose Tissue and Skeletal Muscle

FGF21 stimulates insulin-mediated glucose uptake into adipose tissue (43, 95, 225) in part by increasing expression of the glucose transporter GLUT1 (43, 226). In cultured skeletal muscle myotubes, elevated insulin stimulated FGF21 expression, and FGF21 facilitated insulin-dependent energy uptake by upregulating GLUT1 (227). FGF21 also stimulates adipose tissue uptake of fatty acids derived from lipoproteins (i.e., VLDL and chylomicron) (228), which together with increased glucose uptake functions to direct energy to adipose tissue and effectively redirect calories away from the liver. FGF21 signaling suppresses adipose tissue lipolysis (94, 229), particularly in the fed state (230), and promotes adipocyte lipid homeostasis by ameliorating lipogenesis-associated ER stress (231). FGF21 also increases energy expenditure in mice *via* UCP1-dependent (232) and UCP1-independent (233) mechanisms, which include browning of white adipose tissue by upregulating PGC1α (234). Together, these effects on adipose tissue and skeletal muscle contribute to the role of FGF21 in mediating protection against metabolic disease in numerous models (230, 235). In diet-induced obese mice, exogenous FGF21 decreases body weight and adiposity while improving whole-body insulin sensitivity and lowering serum glucose, insulin, triglycerides, and cholesterol (129, 235, 236). Similar improvements of insulin sensitivity and glycemic control, have been observed in obese non-diabetic, or diabetic non-human primates (44, 183, 237–239). Consistent with these beneficial effects being mediated through adipose tissue, lipodystrophic mice with severely depleted adiposity, or mice with adipose-specific deletion of FGFR1 have blunted glycemic responses to FGF21 (240, 241).

In addition to directly enhancing energy uptake in adipose tissue, FGF21 potently stimulates secretion of adiponectin from adipocytes (44, 100, 225, 236, 242, 243). Increased levels of adiponectin in non-human primates and humans with metabolic disease (type 2 diabetes, obesity and NASH) have been consistently observed following treatment with various FGF21 analogs (183, 244–246). As an adipokine, adiponectin signals from adipose tissue to distal tissues including liver (247). Adiponectin has demonstrated antidiabetic and insulin sensitizing actions (248), in part by suppressing endogenous glucose production (249) and enhancing peripheral fat uptake (250) by increasing lipoprotein lipase activity and enhancing VLDL catabolism (251).

Consistent with improving uptake of energy by peripheral tissues, adiponectin suppresses hepatic steatosis (252) and inflammation (253) in animal models. Based on *in vitro* models of NASH, adiponectin may also exert beneficial effects by acting directly on the liver to reduce hepatic stellate cell activation and migration (254–256). Adiponectin’s biological effects in the liver of NASH patients may be reduced, since expression of its receptors is lower than in liver of patients with simple steatosis (257). In summary, FGF21 enhances peripheral uptake of energy both directly through FGFR-mediated pathways, and indirectly *via* increased adiponectin.

### Fibroblast Growth Factor 21 Ameliorates Dyslipidemia

The earliest and most consistent preclinical observation with FG21 was its amelioration of dyslipidemia. Elevations in plasma LDL-cholesterol, non-HDL-cholesterol, ApoB, and triglycerides are well-established, independent, causal risk factors for cardiovascular disease and major adverse cardiovascular events (258). In diabetic, obese or MCD-induced NAFLD non-human primates, FGF21 (44) or FGF21 analogs (237) robustly reduce serum triglycerides, LDL-cholesterol, and VLDL-cholesterol while increasing HDL-cholesterol. Conversely, knockdown of FGF21 in mice fed a ketogenic diet significantly increases serum lipemia and cholesterolemia (87). The consistent, robust effects of FGF21 analogs are described in greater detail below.

## Fibroblast Growth Factor 21 Protects Other Organs Stressed by Chronic Energy Excess and Metabolic Dysfunction

### Pancreas

By sensitizing adipose tissue and skeletal muscle to insulin, FGF21 reduces demand for insulin secretion, in turn lessening demand for energy generation by β cells. FGF21 also improves β cell homeostasis by promoting autophagy and resistance to ER stress (259, 260). In obese or diabetic mice, FGF21 restores the insulin secretion capacity of β cells *in vivo* (261) *via* several pathways: first, by activating AMPK signaling and autophagy (262); second, by reducing the accumulation of lipotoxic lipids in the pancreas by upregulating expression of *CPT1* (which facilitates oxidation of long-chain fatty acids) and downregulating the expression of lipogenic genes *SREBF1* and *FASN* (263); third, by restoring the expression of core pancreatic genes including *PDX1, INS* (insulin), and *MafA* (264); and finally, by reducing immune cell infiltration and activation within islets (190).

FGF21 also protects the exocrine pancreas from pancreatitis induced by cerulein, associated with accumulation of reactive oxygen species, immune infiltration, and exocrine dysfunction (265). *FGF21* mRNA and protein expression is induced within hours of cerulein-induced supraphysiological exocrine secretion, consistent with an adaptive stress response program (221). Transgenic expression of *FGF21* in pancreatic acini reduces tissue damage, lipid accumulation, and fibrosis induced by cerulein, all of which were exacerbated in animals lacking FGF21 (221, 266). Further, because the functional cells of the pancreas (both exocrine acini and endocrine islets) are highly secretory, FGF21 plays a protective role by enhancing the response to ER stress *via* activation of the UPR (189, 259). By restoring protein folding homeostasis and secretory enzyme trafficking, FGF21 enhances digestive enzyme secretion without increasing protein synthesis (259). These protective responses are not limited to cerulein-induced pancreatitis, but are also observed in mouse models of alcohol-induced pancreatitis and obstructive pancreatitis (189). Together, these stress-responsive, anti-inflammatory, and anti-fibrotic actions of FGF21 preserve endocrine and exocrine function.

### Cardiac and Skeletal Muscle

As with other cell types, FGF21 protects myocytes under cell stress, particularly ER stress and oxidative stress. Because protein chaperone function requires maintenance of intracellular calcium homeostasis, overloaded myocytes are susceptible to ER stress (267). Increased cytoplasmic calcium levels in cardiomyocytes, arising from chronic overloading or mitochondrial dysfunction, are associated with altered protein folding, ER stress, and induction of FGF21 (113).

By mediating expression of the oxidative stress response genes, FGF21 also protects cardiomyocytes from ischemic cell death (268) and oxidative injury caused by LPS-induced inflammation (269). In addition, FGF21 has been reported to protect the heart from inflammation and fibrosis caused by high-fat diet-induced cardiac steatosis (154). Consistent with this, FGF21 is induced by lipid accumulation in cardiomyocytes (112).

### Nervous System

The cytoprotective effects of FGF21, whether direct or indirect, are also evident in the nervous system, where FGF21 treatment attenuates neuroinflammation, neuronal oxidative stress, and neurodegeneration and preserves cognitive function in diabetic mice and high fat fed rats (270, 271). As in other tissues, FGF21 exerts anti-inflammatory effects by suppressing microglial NF-κB signaling, thereby attenuating LPS-induced inflammatory cytokine expression (272). FGF21 also reduces mitochondrial stress in neurons *in vitro* (273) and *in vivo* (271), and improves pathology in mouse models of Parkinson’s (274) and Alzheimer’s disease (275).

## Fibroblast Growth Factor 21 Modulates Food Preference and Fuel Utilization

FGF21 acts *via* three distinct mechanisms to modulate whole-body metabolism. It modifies dietary preferences, regulates metabolism at the level of individual organs, and integrates whole-body metabolism of different substrates.

Firstly, modification of dietary preferences by FGF21 are mediated by behavioral changes consistent with altered taste preference in preclinical models and in humans. Consumption of sucrose by both mice and humans induces FGF21, which serves as a negative feedback mechanism to inhibit consumption (82, 276). In mice, *FGF21* deletion increases sucrose intake, whereas FGF21 administration or overexpression suppresses consumption of sweetened food and beverages (276, 277). These actions appear to be mediated by β-Klotho-expressing glutamatergic neurons in the ventromedial hypothalamus (276) and paraventricular nucleus (278). FGF21 administration to non-human primates also reduces sweet preference, demonstrating a conserved effect on macronutrient preference (277). FGF21 reduces murine preference for ethanol while suppressing dopamine signaling (277). Conversely, a putative loss-of-function *FGF21* allele in humans is associated with greater intake of alcohol (82), preference for sweet foods (279), and altered macronutrient intake (280), but has no effect on total caloric consumption. Additionally, a genome-wide meta-analysis also linked a *KLB* genetic variant with altered alcohol consumption (281).

Secondly, FGF21 appears to modulate substrate utilization by individual organs. For example, it appears to switch cardiomyocyte energy production from glucose utilization to fatty acid oxidation in mice, dependent on *UCP2* induction (282). In myocytes isolated from myopathy patients with an iron-sulfur cluster deficiency, elevated FGF21 expression and secretion correlate with increased expression of ketogenic enzymes (111).

Thirdly, protein restriction in humans (283) and rodents (84), particularly with concomitant high-carbohydrate dietary content (284), increases circulating FGF21. Protein restriction may contribute to perturbations in cellular protein homeostasis, resulting in GCN2-mediated, ATF4-dependent activation of FGF21 expression (84). Higher levels of FGF21 may help to rebalance amino acid availability by enhancing TFEB-dependent lysosomal biogenesis, thereby enabling turnover of proteins within tissues and organs. On a high-fat diet, expression of FGF21 increases whole-body energy expenditure and increases carbohydrate, relative to fatty acid, catabolism (223). Similarly, in diabetic mice, a PEGylated FGF21 analog increases the contribution of glucose to whole-body energy consumption, potentiating glycemic control without affecting body weight (285). On the other hand, mice on a control diet with transgenic overexpression of FGF21 significantly increase ketogenesis in the fed state, consistent with enhanced fat oxidation (89, 286).

In mice, FGF21 appears to improve glycemic control and lipid homeostasis *via* centrally-mediated pathways (287, 288). For example, selective knock out of β-Klotho in regions of the central nervous system abolishes induction of adipose tissue browning, while central infusion of FGF21 increased energy expenditure (289). While endogenous FGF21 is detectable in human cerebrospinal fluid at much lower concentrations than plasma FGF21, it is positively correlated with adiposity (290). However, the extent of FGF21’s centrally mediated effects remains to be determined in humans. For example, while FGF21 stimulates sympathetic activation in mice, elevations in cortisol that would suggest hypothalamic-pituitary-adrenal activation have not been observed in monkeys (239). In mice, the bulk of the metabolic improvement seen upon FGF21 treatment derives from centrally mediated weight loss, whereas FGF21 analogs elicit limited weight loss in humans. Overall, these differences suggest the contributions of FGF21’s diverse metabolic effects vary across species.

In summary, suppression of dietary intake of fructose and sucrose by FGF21 could both reduce liver fat and maintain it at a lower level. In addition, rebalancing of substrate utilization at both organ and whole body level by FGF21 should contribute to improving and maintaining whole body metabolic homeostasis.

## Fibroblast Growth Factor 21 Analogs: Clinical Experience in Patients with Metabolic Disease

**Figure 3:**
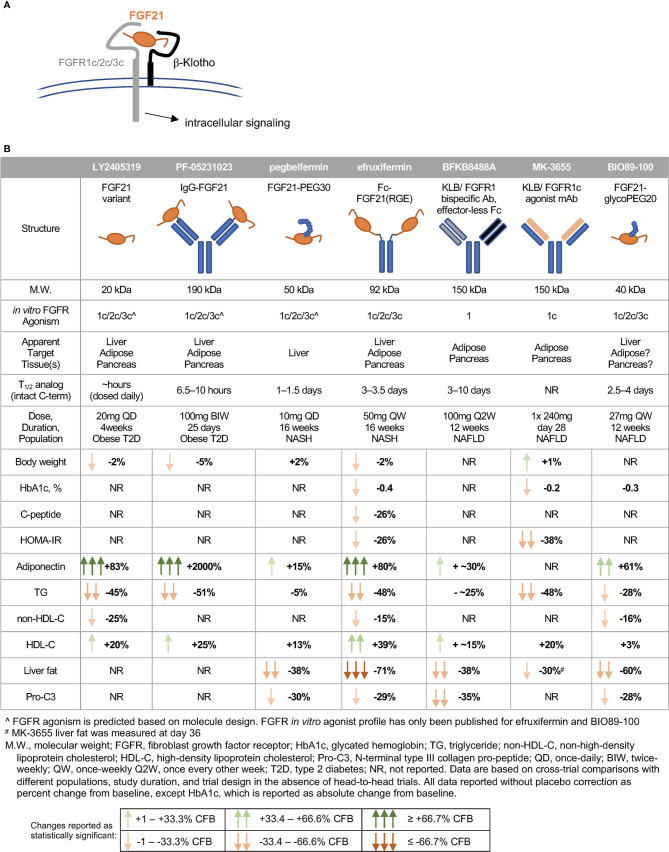
FGF21 analogs demonstrate non-overlapping pharmacological effects in humans. **(A)** FGF21’s C-terminal domain binds -Klotho, enabling N-terminal residues to interact with the c-isoform of the cognate FGFR. **(B)** The clinical pharmacodynamic profile of an FGF21 analog is determined by intrinsic potency as an agonist of FGFR1c/2c/3c, pattern of expression of FGFRs in target tissues, and concentration of intact FGF21 analog at each target tissue/FGFR.

In humans, FGF21 analogs have recapitulated many of the lipid effects and some of the glycemic effects observed in preclinical species, though notably not with all compounds tested (**Figure 3B**). A glycosylated FGF21 variant, LY2405319, maintains the molecular size of endogenous FGF21 but improves its thermal stability and reduces aggregation by engineering an additional disulfide bond and a small number of point mutations into the molecule (291). While the half-life of LY2405319 was not reported, the fact that it was administered daily, coupled with the lack of half-life-extending modifications, suggest a short half-life. Notably, the C-terminal FAP cleavage site remains unmodified, suggesting that LY2405319 is likely susceptible to cleavage and inactivation in human serum *via* loss affinity for KLB (**Figure 3A**). Daily administration of LY2405319 to type 2 diabetes patients for 4 weeks significantly reduced triglycerides by about 45% and LDL-cholesterol by 20–30% from baseline, while increasing HDL-cholesterol by 15–20% and adiponectin by up to 80% (244). LY2405319 administration also reduced levels of ApoB from baseline by up to 20% and ApoC-III by up to 35%, consistent with a shift to a less atherogenic lipoprotein profile. There were slight but not statistically significant decreases in body weight, fasting insulin, and fasting glucose from baseline compared to placebo.

Another analog, PF-05231023, is a 190 kDa molecule consisting of human FGF21 covalently linked to each Fab region of a humanized IgG1κ mAb scaffold, resulting in a 2:1 stoichiometry of FGF21 molecules per scaffold (292). In type 2 diabetes patients, twice-weekly administration of PF-05231023 for 4 weeks reduced body weight by about 5%, triglycerides by about 50%, and LDL-cholesterol by about 20% from baseline, while increasing HDL-cholesterol by about 15% and adiponectin 15- to 20-fold (183). However, modest decreases in glucose, insulin, and HOMA-IR (homeostatic model assessment for insulin resistance) did not reach statistical significance (183). Weekly administration of the same molecule for 4 weeks in a second study with obese hypertriglyceridemic subjects demonstrated similar effects on triglycerides, HDL- and non-HDL cholesterol, and adiponectin, but without affecting body weight (293). Although PF-05231023 demonstrated pharmacodynamic effects when dosed weekly or twice-weekly in humans, the unmodified N- and C-termini of the FGF21 moities resulted in a short (approximately 8 h) half-life for the intact C-terminal domain of FGF21 (183). As with LY2405319, it appears unlikely that a high level of agonism of FGF21’s receptors was sustained between doses.

A PEGylated FGF21, pegbelfermin (formerly BMS-986036), consists of a 30 kDa PEG moiety attached to residue 108 of FGF21, yielding a 50 kDa analog (285). One of only two FGF21 analogs tested in a study of biopsy-confirmed NASH patients, pegbelfermin administered daily or weekly for 16 weeks reduced triglycerides by 5% and LDL-cholesterol by about 10% from baseline, while increasing HDL-cholesterol by about 12% and adiponectin by 15% (245). No effects on body weight or glycemic control were reported in this study. While the changes in lipid parameters are smaller compared to those elicited by LY2405319 or PF-05231023, pegbelfermin is the first FGF21 analog to demonstrate beneficial hepatic effects in NASH patients, with daily administration yielding approximately a 30% relative reduction in liver fat content. Pegbelfermin additionally reduced levels of serum alanine aminotransferase (ALT) and Pro-C3 (a marker of fibrogenesis) by up to 30%, consistent with improved hepatic health. Notably, daily dosing of 10 mg pegbelfermin delivered stronger improvements in clinical parameters than weekly dosing of 20 mg pegbelfermin, consistent with a reported intact C-terminal half-life of 1–1.5 days in humans (294). Daily or weekly administration of pegbelfermin for 12 weeks in type 2 diabetes patients recapitulated these modest effects on lipid parameters without altering body weight, and did not elicit clinically or statistically significant changes in markers of glycemic control (295). Because of the location of the PEG moiety and absence of additional modifications at the C-terminus of the FGF21 moiety, pegbelfermin remains susceptible to FAP cleavage and inactivation.

Efruxifermin (formerly AKR-001, AMG 876), the only other FGF21 analog studied in a clinical trial of biopsy-confirmed NASH patients, is a 92 kDa Fc-FGF21fusion protein with a 3–3.5 day half-life (based on intact C-terminal domain of the FGF21 moiety), which is substantially extended relative to the FGF21 analogs described above. This longer half-life results both from endosomal recycling mediated by the neonatal Fc receptor, and from stabilization of the N- and C-termini of the FGF21 moiety. Because the Fc domain is translated directly upstream of a peptide linker and N-terminus of the FGF21 variant, the N-terminus is protected from degradation by dipeptidyl peptidase (DPP) enzymes (296). Protection of the C-terminus is achieved *via* substitution of a glycine for the native proline at residue 171 (P171G) relative to mature (i.e., signal peptide removed) human FGF21, preventing degradation by FAP. In the context of a potential therapy for NASH, stabilization against FAP degradation is noteworthy because expression of FAP is greatly elevated in liver from NASH patients compared to appropriate control liver (297). Two additional substitutions further enhance the *in vivo* activity of efruxifermin: A180E, which increases affinity for KLB, and L98R, which decreases aggregation (238). Efruxifermin retains *in vitro* balanced potency as an agonist of FGFR1c, FGFR2c, and FGFR3c, a characteristic that may be required to elicit maximal effects on liver and extra-hepatic tissues. In a 4-week study in type 2 diabetes patients, weekly administration of up to 70 mg efruxifermin reduced triglycerides by 60–70%, non-HDL-cholesterol by about 35%, fasting insulin about 50%, and fasting glucose about 20% compared to baseline, while increasing HDL-cholesterol by 60% and adiponectin by about 95% (246). Efruxifermin did not significantly alter body weight over this 4-week trial but did significantly reduce the atherogenic lipoproteins ApoB and ApoC-III to a greater extent than did LY2405319 over the same period. Intriguingly, weekly but not every-other-weekly administration of efruxifermin demonstrated for the first time that an FGF21 analog could deliver improved glycemic control in humans, suggesting that the limitations of previous molecules may have been rooted in inadequate exposure. In a subsequent, 16-week study in biopsy-confirmed F1–F3 NASH patients, approximately half of whom were type 2 diabetes patients, efruxifermin improved glycemic control with significant reductions in HbA1c at doses up to 70 mg weekly. Improvements in lipid and lipoprotein profile were also observed, consistent with those seen after 4 weeks’ treatment. Efruxifermin reduced liver fat content by over 70% after 12 weeks dosing. For the first time, an FGF21 analog induced a strong reduction in liver fat that appeared to be associated with resolution of histological features of NASH, including fibrosis (298). An observed rapid and sustained 30% reduction in serum Pro-C3 levels is consistent with preclinical literature demonstrating FGF21’s antifibrotic activity, and with the reduction in Pro-C3 observed upon pegbelfermin treatment. Finally, the longer 16-week dosing revealed efruxifermin-associated weight loss.

A second PEGylated FGF21, BIO89-100, prevents C-terminal degradation by attachment of a PEG moiety to the amino acid residue adjacent to the FAP cleavage site, resulting in a 2.5–4 day half-life in healthy volunteers. This analog retains balanced *in vitro* potency at FGFR1c, FGFR2c, and FGFR3c (299). In a multiple-ascending dose study in NAFLD subjects, about 20% of whom had biopsy-confirmed NASH, weekly or every-other-weekly administration of BIO89-100 for 12 weeks reduced serum triglycerides by 18–28%, reduced liver fat by 36–60%, and increased adiponectin by 23–61% relative to baseline (299). Notably, only the highest dose of BIO89-100 administered weekly appeared to elicit strong pharmacodynamic effects mediated *via* FGFR1c activity in adipose tissue, i.e., significantly increasing systemic levels of adiponectin. Every-other-weekly administration of BIO89-100 did not improve markers of glycemic control, with only the highest once-weekly dose showing a trend towards improvements (299). The smaller magnitude of effect on liver fat, HbA1c and lipoprotein profile for BIO89-100 with every-other-weekly dosing appears to limit the value of extending the dosing regimen (299).

Two antibody-based approaches have been developed to mimic FGF21 signaling through FGFR1c only. BFKB8488A is a humanized bispecific antibody that specifically activates the FGFR1/β-Klotho complex (300). In a 12-week study in NAFLD patients, every-other-weekly administration of up to 100 mg BFKB8488A, the highest adequately tolerated dose, reduced liver fat by about 40%, serum triglycerides and Pro-C3 by up to 25 to 30% from baseline, while increasing adiponectin by up to 40% and HDL-cholesterol by up to 20% (301). A parallel study of BFKB8488A in type 2 diabetes patients recapitulated improvements in HDL-cholesterol, triglycerides, and adiponectin, but did not demonstrate improvements in HbA1c, fasting glucose, or fasting insulin (302). Changes in body weight were not reported in either study.

Another monoclonal antibody targeted to β-Klotho and FGFR1c, MK-3655 (formerly NGM313), has been tested in a single-dose study by comparison with pioglitazone (303). It has an extended half-life, and appeared to improve glycemic control in obese, non-diabetic subjects. Liver fat content was reduced by 30–35% relative to baseline at day 36 after administration, while serum triglycerides and LDL-cholesterol were reduced and HDL-cholesterol increased (303). As observed with pioglitazone, MK-3655 was associated with statistically significant weight gain from baseline to day 36. Based on these data, monthly dosing may be achievable with MK3655.

### Factors Underlying Differences Between Fibroblast Growth Factor 21 Analogs

While LY2405319 (244), PF-05231023 (183), efruxifermin (246), BFKB8488A (301), and MK-3655 (303) were observed to ameliorate dyslipidemia, with strong reductions in triglycerides and increases in HDL-cholesterol, only efruxifermin and MK-3655 have so far demonstrated the potential to increase insulin sensitivity and to improve glycemic control. Further, while pegbelfermin (245), BFKB8488A (301), BIO89-100 (299), MK-3655 (303), and efruxifermin (298) have all been associated with reductions in ALT, Pro-C3, and hepatic fat fraction, the observed magnitude of liver fat reduction was greatest following efruxifermin treatment. Several structural parameters differentiate these molecules that may explain the different clinical profiles (**Figure 3B**).

Maximal reduction of liver fat appears to require both inhibition of hepatic *de novo* lipogenesis and suppression of adipose tissue lipolysis (51, 52). For FGF21 analogs, this is likely to require balanced agonism of FGFR1c, FGFR2c and possibly FGFR3c. The almost 2-fold greater reduction in liver fat with efruxifermin than with FGFR1-selective BFKB8488A is consistent with this argument, as FGFR1c agonism would mainly reduce steatosis by suppressing adipose tissue lipolysis. While the *in vitro* potency of pegbelfermin as an agonist of FGFR1c, FGFR2c and FGFR3c has not been reported, the modest induction of adiponectin in humans suggests that it exerts minimal action through adipose tissue FGFR1c in humans. As a result, suppression of adipose tissue lipolysis by pegbelfermin is likely limited. Consistent with this, pegbelfermin reduced liver fat by about half the extent of efruxifermin, and serum triglyceride by about 10%, compared to about 30–50% for other FGF21 analogs, including FGFR1c selective antibodies. BIO89-100, on the other hand, appears to deliver relative hepatic fat loss in between efruxifermin and pegbelfermin, with the highest dose reducing fat content by 60% in NAFLD patients, and all other doses reducing fat content by 36–50% (299). While BIO89-100 has been reported to be a balanced agonist of FGF21’s receptors *in vitro*, the smaller magnitude of effect on adiponectin compared to efruxifermin suggest that BIO89-100 may not have maximally stimulated FGFR1c signaling in adipose tissue at the doses tested in humans (**Figure 3B**). Notably, comparisons of these analogs are limited because efruxifermin and pegbelfermin have been evaluated in patients with confirmed NASH, while BIO89-100 has been studied in patients with less severe NAFLD.

FGF21’s effects on glycemic control and insulin sensitivity are likely mediated predominantly by FGFR1c signaling in adipose tissue, which should normally serve as one of the body’s main depots for excess calories in response to post-meal insulin. To enhance insulin sensitivity and improve glycemic control, it appears that FGF21 analogs must maintain FGFR1c activation in adipose tissue above a threshold level throughout the inter-dose interval. For example, 70 mg efruxifermin dosed weekly, corresponding to two pharmacokinetic half-lives, appeared to improve HOMA-IR, fasting glucose, and fasting insulin in type 2 diabetes patients (246). By comparison, every-other-weekly dosing of 140 mg achieved equivalent total exposure over 4 weeks, but with four pharmacokinetic half-lives between doses, did not appear to improve glycemic control, despite comparable effects on serum triglycerides and lipoproteins. Consistent with this, the FGR1c-selective MK-3655, with the long half-life of a monoclonal antibody, is the only other FGF21 analog that appears to improve glycemic control, though repeat-dose data has not been reported. The β-Klotho/FGFR1 bispecific agonist antibody, BFKB8488A, failed to improve glycemic control, suggesting that there may have been insufficient agonism of FGFR1c throughout the every-other-weekly dosing interval.

### Differentiation Between Analogs of Fibroblast Growth Factor 21 and Fibroblast Growth Factor 19

An FGF19 analog, aldafermin (formerly NGM282, M70) has a five-amino acid deletion and three amino acid substitutions in the N-terminal region of human FGF19 (304). This engineering was necessary to reduce the proliferative effect of native FGF19 on hepatocytes, mediated *via* signaling through FGFR4—the only FGF receptor at which FGF19 and FGF21 differ in agonist activity (305). When administered to patients with NASH for 12 weeks, aldafermin (up to 6 mg daily) reduced liver fat content by up to 60% relative to baseline, reduced ALT by about 50%, and decreased Pro-C3 by about 20–25% (31). However, consistent with FGFR4-mediated suppression of CYP7A1, which catalyzes the rate limiting step of cholesterol metabolism to bile acid, aldafermin significantly increased LDL-cholesterol and non-HDL cholesterol without exerting a consistent effect on insulin, glucose, or HbA1c. In a longer-term 24-week study in NASH patients, daily administration of 1 mg aldafermin led to an approximately 30% placebo-adjusted relative reduction in liver fat while reducing ALT by about 45% from baseline. However, statin co-administration was required in more than 95% of treated subjects to mitigate the increase in LDL-cholesterol, which was also observed previously at higher doses (306). This study confirmed that aldafermin treatment did not improve glycemic control. The lack of effect of FGF19 on glycemic control is consistent with preclinical studies. For example, selective FGFR4 agonism, either peripherally or in liver, does not affect fasting glucose in obese diabetic mice, but increases triglycerides and total cholesterol (307, 308). Conversely, either germline knockout or antisense oligonucleotide knockdown of *FGFR4* expression in mice enhances hepatic fat oxidation, glucose metabolism, and insulin sensitivity (308–310) while reducing hepatic steatosis (308, 309). While these metabolic effects were likely to have been mediated by compensatory upregulation of FGF19 and/or FGF21 (308), they were clearly independent of FGFR4 agonism, instead being mediated by agonism of FGFR1c, 2c or 3c.

As elevated bile acids are associated with fibrotic liver disease, and FGF19–FGFR4 signaling suppresses bile acid synthesis, it has been posited that reducing bile acids may deliver anti-fibrotic actions (311). However, anti-fibrotic actions of FGF19 signaling do not appear to depend on FGFR4 signaling, based on three lines of evidence. First, both FGF21 (216, 217) and FGF19 (312) directly inhibit HSC activation and proliferation *in vitro*. Second, FGF21 suppresses TGF-β release from pro-inflammatory macrophages *in vitro* (177), which would be expected to reduce Kupffer cell activation of HSCs *in vivo*. While a similar effect does not appear to have been reported for FGF19, this indirect anti-fibrotic effect of FGF21 suggests that FGFR4 is unnecessary for inhibition of Kupffer cell-HSC signaling. Third, analogs of FGF21 and FGF19 appear to reduce markers of liver injury (ALT) and fibrosis (Pro-C3) to a similar extent in humans (245, 306), consistent with FGFR4 agonism not being required *in vivo*, while being associated with an undesirable increase in LDL-C.

### Safety and Tolerability of Fibroblast Growth Factor 21 Analogs in Clinical Trials

Preclinical studies have suggested that FGF21 increases tone of the hypothalamic-pituitary-adrenal (HPA) axis (287, 288). However, whether FGF21 analogs modulate the HPA axis in humans remains incompletely characterized. Increased levels of corticotropin-releasing hormone or corticosterone/cortisol with FGF21 or FGF21 analog treatment have been reported in rodents, but not in non-human primates nor in any human studies (183, 293, 313). Likewise, reports of FGF21 increasing water intake and blood pressure in rodents, potentially by stimulating sympathetic pathways (313, 314) do not appear to translate consistently to humans, with one study (293) but no others (183, 244–246, 315) suggesting an effect. The lack of effect is consistent with blood pressure phenotypes of humans with *FGF21* genetic variants. The minor A allele of rs838133 corresponds to a putative *FGF21* loss-of-function allele, which is associated with 0.29 mmHg higher systolic blood pressure per allele (279). This study also identified a rare variant encoding a truncated, putatitive loss-of-function FGF21 protein, associated with 7.5 mmHg higher blood pressure. Additionally, administration or overexpression of FGF21 reduces blood pressure in various preclinical models of hypertension (316–318). Overall, the preclinical and clinical data do not support a likely hypertensive effect of FGF21 analogs downstream of increased sympathetic tone in humans.

The apparent differences between rodents and primates may reflect differences in blood-brain barrier permeability across species (319). Mice in particular (320) may be more sensitive to systemically administered FGF21 and FGF21 analogs than humans. Larger molecules based on antibody scaffolds and fragments, including PF-05231023 (190 kDa), BFKB8488A and MK-3655 (150 kDa), and efruxifermin (92 kDa) may be less likely to cross the blood-brain barrier at therapeutic doses, but their tissue distribution has not been described. Emerging clinical observations with smaller FGF21 analogs including pegbelfermin (50 kDa) and BIO89-100 (40 kDa), may provide insights into effects possibly mediated *via* the central nervous system.

While some preclinical studies with FGF21 and FGF21 analogs have suggested effects on bone turnover, clinical data have not demonstrated a consistent effect. One group demonstrated that FGF21 increased bone resorption and decreased bone mass in mice (321), in part *via* induction of hepatic insulin-like growth factor binding protein-1 (IGFBP1) (322). However, a more recent preclinical study did not find evidence of FGF21-induced bone loss in a diet-induced model of obesity (323). While the first clinical trial of PF-05231023 demonstrated a decrease in markers of bone formation and an increase in markers of bone resorption, this was accompanied by a significant reduction in body weight. A subsequent study of PF-05231023 did not demonstrate weight loss, and there were no changes observed in markers of bone formation or resorption, IGFBP1–3, or free IGF1 (293). No subsequent clinical studies of FGF21 analogs in adults have demonstrated a significant effect on bone density or markers of bone resorption and formation in adults (245, 246, 295). Notably, while markers of type I collagen synthesis and degradation are frequently used to indicate changes in bone turnover, they may also reflect changes in liver fibrogenesis or fibrinolysis (324, 325). Overall, the weight of evidence to date does not support a role for FGF21 in mediating bone remodeling in adult humans.

The most prevalent and consistent dose-limiting side effects observed across studies of FGF21 analogs in patients with metabolic disease are gastrointestinal (GI), including mild-to-moderate nausea and diarrhea (183, 245, 246, 298, 301). These side effects were also seen in trials of the FGF19 analog, aldafermin (31, 306). However, preclinical studies of these various endocrine FGF analogs in rodents and monkeys did not reveal GI symptoms, therefore the potential mechanisms underlying adverse GI effects have not been investigated (**Table 2**). It is possible that FGF21 exerts some direct or indirect effect on the GI tract, but experiments directly testing this hypothesis have not been reported to date.

**Table 2:** Readthrough from preclinical to clinical safety and tolerability for FGF21 analogs.

Class	Signs/symptoms	Observed in rodents?	Observed in monkeys?	Observed in humans?
GI	Nausea, diarrhea, vomiting altered appetite	No ↑	No ↓	Yes ↓ and ↑
Altered bone homeostasis	Increased bone turnover markers	Yes	No	PF-05231023 only
Activation of HPA axis	Increased cortisol Increased water intake/urine output Increased blood pressure	Yes Yes Yes	No No No	No PF-05231023 only PF-05231023 only

GI, gastrointestinal; HPA, hypothalamus-pituiary-adrenal; ↑, increase or ↓, decrease in one or more studies.

Whether GI tolerability affects patient adherence to or uptake of FGF21-based therapies in NASH remains to be seen. However, precedent suggests that mild to moderate adverse GI events are not a major barrier to uptake of a promising metabolic therapy. While GLP-1 analogs for treatment of type 2 diabetes cause mild-to-moderate nausea and vomiting in a significant proportion of patients, their benefits in terms of weight loss and improved glycemic control have allowed the class to overcome these tolerability concerns and become one of the most-prescribed classes of type 2 diabetes medications in the US and Europe (326). Second, up-titration regimens for GLP-1 analogs have mitigated adverse GI side effects, reducing their prevalence and severity in both clinical trials and post-marketing surveillance (327). FGF21 analogs have not so far employed up-titration, but given the reportedly mild-to-moderate as well as the transient nature of the nausea and diarrhea, such an approach could reasonably be expected to mitigate the GI side effects.

## Conclusions and Outlook

Despite uninspiring outcomes in clinical trials of the first wave of potential NASH therapies, a spate of promising mid-to-late stage clinical trials has provided a needed morale boost to NASH patients, endocrinologists and hepatologists, and caregivers. The complex pathophysiology of NASH mandates a holistic approach to treating patients, focusing not only on liver histology but also cardiovascular risk and glycemic control, which has proven to be a high bar (**Table 1**).

An FXR agonist, obeticholic acid, is the first molecule to deliver a statistically significant improvement in one of the FDA’s surrogate endpoints for accelerated approval of a treatment for NASH. However, it is associated with a risk of liver injury in patients with primary biliary cholangitis (328), and significantly elevates LDL-cholesterol (30, 32). FGF19, like FXR agonists, has demonstrated potential to improve liver histology, but also exacerbates dyslipidemia already common in NASH patients (306). Inhibitors of acetyl-CoA carboxylase demonstrate a reduction in hepatic fat fraction, but significantly increase serum triglyceride levels (34). PPAR agonists have demonstrated insulin sensitizing effects and some liver-specific improvements, but are associated with increased body weight (329). The GLP-1 analog semaglutide demonstrated a strong ability to resolve NASH, and is associated with weight loss and improved cardiovascular health, but did not statistically significantly improve fibrosis (326). TR-β agonists have demonstrated improvements in liver fat and biomarkers of cardiovascular disease along with significant resolution of NASH, but have not demonstrated a strong anti-fibrotic effect (330). Across the field of potential NASH drugs, each mechanistic class appears to deliver improvements in a subset of liver histology, whole-body metabolism, or cardiovascular risk profile, but not all three. Moreover, some mechanisms worsen cardiovascular risk profile despite improving liver health, which appears incompatible with long-term use in patients with increased cardiovascular risk.

FGF21 analogs therefore represent a promising emerging class of NASH therapeutics, as a number of them have demonstrated pleiotropic effects consistent with improved liver health and whole-body metabolism. Because of an elevated risk of cardiovascular morbidity and mortality in NASH patients prior to advanced fibrosis, as well as hepatic decompensation, HCC, and/or liver failure, therapeutic options must address the complex pathophysiology of this disease. Analogs of FGF21 that are able to maintain balanced and sustained agonism of FGFR1c, FGFR2c, and FGFR3c throughout the inter-dose interval appear to show the most promising pharmacological profiles, with among the largest reported decreases in liver fat of any therapeutic mechanism tested in NASH patients, and with encouraging signs of resolving NASH histology and fibrosis after relatively short-term treatment. FGF21 analogs with balanced receptor agonism and sustained exposure have also been shown to improve glycemic control, ameliorate dyslipidemia and reduce body weight. Should this pharmacological profile be confirmed in late-stage clinical studies, optimized FGF21 analogs have the potential to not only resolve liver pathology, but also to reduce risk of cardiovascular morbidity and mortality, and incidence of organ damage associated with inadequate glycemic control.

## Author Contributions

ET and TR wrote the first draft, edited, and wrote the final draft of this article. All authors contributed to the article and approved the submitted version.

## Conflict of Interest

ET is an employee and shareholder of Akero Therapeutics. TR is a co-founder, employee, and shareholder of Akero Therapeutics, and a shareholder of Pfizer.
